# Finding Structure in Time: Visualizing and Analyzing Behavioral Time Series

**DOI:** 10.3389/fpsyg.2020.01457

**Published:** 2020-07-24

**Authors:** Tian Linger Xu, Kaya de Barbaro, Drew H. Abney, Ralf F. A. Cox

**Affiliations:** ^1^Department of Psychological and Brain Sciences, Indiana University, Bloomington, IN, United States; ^2^Department of Psychology, The University of Texas at Austin, Austin, TX, United States; ^3^Department of Psychology, Center for Cognition, Action & Perception, University of Cincinnati, Cincinnati, OH, United States; ^4^Department of Psychology, University of Groningen, Groningen, Netherlands

**Keywords:** time series analysis, data visualization, burstiness, cross recurrence quantification analysis, Granger causality, high-density behavior data

## Abstract

The temporal structure of behavior contains a rich source of information about its dynamic organization, origins, and development. Today, advances in sensing and data storage allow researchers to collect multiple dimensions of behavioral data at a fine temporal scale both in and out of the laboratory, leading to the curation of massive multimodal corpora of behavior. However, along with these new opportunities come new challenges. Theories are often underspecified as to the exact nature of these unfolding interactions, and psychologists have limited ready-to-use methods and training for quantifying structures and patterns in behavioral time series. In this paper, we will introduce four techniques to interpret and analyze high-density multi-modal behavior data, namely, to: (1) visualize the raw time series, (2) describe the overall distributional structure of temporal events (Burstiness calculation), (3) characterize the non-linear dynamics over multiple timescales with Chromatic and Anisotropic Cross-Recurrence Quantification Analysis (CRQA), (4) and quantify the directional relations among a set of interdependent multimodal behavioral variables with Granger Causality. Each technique is introduced in a module with conceptual background, sample data drawn from empirical studies and ready-to-use Matlab scripts. The code modules showcase each technique’s application with detailed documentation to allow more advanced users to adapt them to their own datasets. Additionally, to make our modules more accessible to beginner programmers, we provide a “Programming Basics” module that introduces common functions for working with behavioral timeseries data in Matlab. Together, the materials provide a practical introduction to a range of analyses that psychologists can use to discover temporal structure in high-density behavioral data.

## Introduction

Our title was inspired by a highly influential paper by Jeffrey L. Elman highlighting the importance of characterizing the temporal structure of behavior for understanding human cognition (Elman, 1990). We believe this is even more true for studying human development. All forms of behavior are organized as cascades of real-time events (Spivey and Dale, 2006; Adolph et al., 2018). The micro-dynamics of infants’ behaviors and their interactions with the world shape their longitudinal trajectories across domains, from motor and language development to socio-emotional development and psychopathology (Thelen, 2000; Adolph and Berger, 2006; Masten and Cicchetti, 2010; Landa et al., 2013; Blair et al., 2015; West and Iverson, 2017). By studying behavior as it unfolds over time, we are able to reveal rich source of information about its dynamic organization, origins, and development (Bakeman and Quera, 2011).

In the past two decades, technological advances in sensing and mobile computing have provided researchers with new ways to collect behavioral data at a fine temporal scale both in and out of the laboratory (de Barbaro, 2019). This has led to the curation of massive multimodal corpora of behavior (Yang and Hsu, 2010; Franchak and Adolph, 2014; Smith et al., 2015; Matthis et al., 2018). Leveraging these massive new datasets to characterize the complex processes of human behavior presents outstanding opportunities, as well as challenges for psychologists in all fields.

Analysis of these rich corpora of behavioral data faces three main challenges. The first challenge is that our world is profoundly multimodal (Smith and Gasser, 2005; Kolodny and Edelman, 2015). Behaviors are organized with multiple time-locked sensory-motor systems which influence each other simultaneously (Garrod and Pickering, 2009; Louwerse et al., 2012; Fusaroli and Tylén, 2016). In daily social interactions, people communicate via gaze, speech, facial expression, gesture and even body movements (Vinciarelli et al., 2009; Knapp et al., 2013; de Barbaro et al., 2016b). At every moment during an interaction, interlocutors respond to the multimodal behavioral signals from one another, make adjustments, and influence one another in real time. This poses a major methodological challenge to researchers interested in the complex structure of activity within and between individuals. Specifically, how should the directional influence from one behavioral variable to another within a system be quantified when multiple variables are interdependent on each other?

Second, behaviors occur at and unfold across many distinct interconnected timescales (Ballard et al., 1997; Wijnants et al., 2012a; Abney et al., 2014; Fusaroli et al., 2015; Darst et al., 2016; Den Hartigh et al., 2016). Facial expressions, eye gaze shifts and bursts of laughter occur at short timescales, often lasting less than one second (Kendon, 1970; Hayhoe and Ballard, 2005; de Barbaro et al., 2011; Knapp et al., 2013). At longer timescales, these micro behaviors are organized into more extended episodes of interaction. For instance, conversations occur at timescales of minutes or hours, contributing to language development (Cox and van Dijk, 2013), whereas friendships can last years or decades (Demir, 2015). Behaviors at different temporal scales have their own emergent properties with hierarchical relations. For example, reading a bedtime story is composed of a sequence of activities including choosing a book, reading the book, and finishing the story with a good night kiss, coordinated through a complex set of embodied vocal and attentional exchanges (Sénéchal et al., 1995; Rossmanith et al., 2014; Flack et al., 2018); walking emerges from tens of thousands of steps and hundreds of failed attempts (Adolph et al., 2011; Adolph and Tamis-Lemonda, 2014); a secure (or insecure relationship) is formed by numerous interactions, play activities and conversations spanning of days, months or even years (Granic and Patterson, 2006). In order to adequately describe and model the multi-scaled nature of human behavior, we need hierarchical methods that can identify or integrate shifts in activity across these temporal scales, for example, to characterize how interaction patterns change during joint activity over the first year (de Barbaro et al., 2013; Rossmanith et al., 2014), or how micro-dynamics of facial activity become organized in laughter vs. crying (Messinger et al., 2012).

The last challenge is that changes in behavioral time series are often non-linear (Carello and Moreno, 2005; Dale and Kello, 2018). We know that behaviors change over time, yet we often ignore the fact that the rate of those changes may also vary from time to time. Conventional statistical methods work under the assumption that variations in the collected behavioral data are stationary across time. However, this assumption does not hold in a variety of complex environments and thus the results are lost in the averaging process. For example, Tamis-LeMonda et al. (2017) recorded the speech generated by the parents in a 45-min session of child-parent naturalistic play session and calculated the word-type over word-token ratio as a measure for speech complexity. The word type token ratio was computed both over the course of the entire play session and within each minute from the start to end. The results showed large temporal fluctuation both in raw speech quantity and word type token ratio over the course of the play session. Parental speech is not uniform; rather, the structure and complexity of the speech is dependent on real-time play content. Aggregative methods, which assume that changes in behaviors and interactions are stationary, are thus likely to miss the true complexity of dynamic activity. As more and more studies aim to discover important questions in more naturalistic experimental settings, we need methods that can reveal non-linear changes in human behaviors across temporal scales.

Overall, it can be said that modern behavioral science faces a “curse of dimensionality” (Bellman, 1961): multimodal, high-density temporal datasets that are collected in relatively unconstrained settings lead to an analysis overload. These vast amounts of data have yet to be truly leveraged to their full advantage (Aslin, 2012; Yu et al., 2012). Critically, there are few if any domain-specific analytical tools that can characterize high-density multimodal dynamics of human activities using such emerging datasets. Indeed, the complexities of these systems mean that no single tool will work to quantify the complex behaviors and uncover intriguing patterns (Gnisci et al., 2008). In this paper, we provide readers a practical introduction to an ensemble of four analytic techniques to characterize the temporal structure of high-density behavioral data. Each technique is introduced in a module associated with sample data and code available on Github, https://github.com/findstructureintime/Time-Series-Analysis (Xu et al., 2020), as well as conceptual material in the manuscript text, including an explanation of the technique and its application to the showcase example data provided in the code module.

The techniques covered in the modules can be used to characterize distinct aspects of the temporal structure of behavioral data. The first module provides a step-by-step “programming basics” tutorial that introduces users to common behavioral timeseries data as well as scripts necessary to import and manipulate these data. The goal is to provide novice users the necessary scaffolding to begin to work with behavioral timeseries data and make sense of the subsequent modules. It also provides scripts for transforming data to and from common data formats used across all modules to facilitate modifying module material to work with user data. The second module focuses on the visualization of raw behavioral data. Visualizations allow researchers to observe the rich dynamics of complex multimodal data across multiple timescales, making structure in activity apparent where it may not be theoretically prespecified. Visualizations can thus suggest the most appropriate variables or analyses as part of a “human-in-the-loop” analysis (Card et al., 1999; Shneiderman, 2002). The third module introduces a way to describe the distributional structure of temporal events: Burstiness calculation. This is a method to quantify the temporal regularity of occurrence of events (Goh and Barabási, 2008). The fourth module explains Chromatic and Anisotropic Cross-Recurrence Quantification Analysis which can be used to characterize coupled non-linear dynamics over multiple timescales. These techniques can reveal different types of recurrent behavioral patterns and can quantify asymmetries in the coupling strength between two temporal variables. The last module introduces Granger Causality as a novel way to quantify the directional relations among multiple behavioral time series. Multiple channels of behaviors are often interdependent with each other. This technique provides a way to investigate the unique influence from one behavioral time series to another while taking all the variables in the system into consideration.

In sum, the techniques introduced in this paper cover a wide range of analysis needs for psychologists dealing with time series data: from visualization to computational analysis; from quantifying distributional regularities to discovering underlying non-linear structures and synchronization patterns; from describing the patterns within one behavioral time series to computing the quantitative directional relations from multimodal behavioral time series. Our goal is that both beginner and experienced programmers will be able to selectively benefit from the provided materials. Novice programmers will benefit most from the modules if they carefully work through the material in Modules 1 and 2 before attempting to run or modify the later modules. More experienced programmers can more selectively focus on the modules of greatest interest to them, modifying scripts to meet their own analysis goals.

All associated scripts can be run and edited using Matlab 2018a and later versions available on Windows, macOS, and Linux operating systems. While Matlab requires a paid subscription, many institutions offer free access to this software. Readers who are not familiar with Matlab are advised to reference the Mathworks website where a complete list of free campus licenses is available. Alternatively, readers can use the open source GNU Octave (version 5.2.0 or later, which runs on GNU/Linux, macOS, BSD, and Windows) to run and modify the scripts.

## Module 1: Time Series Programming Basics

To make our modules accessible to novice programmers, the first module provides a tutorial introducing novice programmers to main temporal data types commonly collected in behavioral science research and basic syntax useful for working with multimodal temporal behavioral data. The module also walks users through importing, manipulating and making simple plots of timeseries data. The goal is to provide users with little to no programming background with relevant programming experience to begin working with their own temporal datasets. While programming expertise is a continuous learning practice that is never truly “complete,” these scripts can serve as a jumping off point by which inexperienced readers can build the skills and confidence necessary to understand and modify the scripts in the subsequent modules to accommodate their own data and research questions.

Additionally, this module provides scripts to transform outputs of annotation software commonly used in developmental science research, such as Elan, The Observer, or Mangold Interact (Noldus, 1991; Wittenburg et al., 2006; Mangold, 2017) into data formats compatible with all subsequent modules. These scripts thus further enable novice programmers to apply subsequent modules to their own data.

### Methods

The most common types of temporal data collected in developmental science research are event data and timeseries data. Event data are those in which each event of interest is indicated by an onset timestamp, an offset timestamp and a third value that represents the behavioral code, i.e., looking at or manipulating a certain target object. Event data are commonly used when indicating discrete behaviors, including for example, sequences of infant gaze or dyadic interaction states. Event data could also include irregularly spaced data such as a list of ecological momentary assessments, paired with the time they are completed. Within the field of developmental science, event data are often generated by human annotation (i.e., labeling) of audio or video records. By contrast, timeseries data are data points sampled at equally spaced intervals in time with a specified sampling rate, such as 10 HZ which means 10 data points are sampled at every 100 ms. Examples of common behavioral timeseries data include frame-by-frame presence or absence of mutual gaze between two interactors, positive or negative affect state recorded every second throughout a 10-min play session or observation of the presence of caregiver’s face in infant’s first person view within every 5 s interval.

Binary spike train data is a specific type of timeseries data in which a ‘1’ represents the onset of an event of interest and a ‘0’ represents instances when an onset did not occur (also known as “point process data”). This type of time series data is used to compute inter-onset intervals, i.e., the duration of time between the onset of consecutive events or to construct likelihood models of an event’s occurrence.

Note that any event sequence data can be transformed into a timeseries. Additionally, timeseries data can be transformed into event data, although for continuous timeseries this may require setting thresholds to “parse” the data into distinct events. Finally, event data can be transformed to binary spike trains by including only the *onsets* of the events in the binary spike train timeseries. This is critical as some analyses require one data format rather than the other. In particular, event data inputs are used in Modules 1 and 2 (Visualization), timeseries data inputs are used in Modules 1, 2 (Visualization) and 4 (Recurrence Quantification Analysis), and binary spike train inputs are used in Modules 3 (Burstiness analysis) and 5 (Granger Causality).

#### Sample Data and Scripts

This module includes seven scripts as well as step-by-step tutorial instructions in the *readme.md*.

##### Data

To introduce readers to timeseries and event data type, this module includes samples of simple data from a study of the development of joint activity, which examined frame-by-frame annotations of all mother and infant gaze and touching behaviors to a set of three available objects (de Barbaro et al., 2016b). Additionally, it includes several sample files exported from the annotation software Mangold (Mangold, 2017) that will be used to practice data import and data format transformation. The files contain multiple dimensions of data, including mother and infant affect events. Users will also create visualizations with this sample dataset in Module 2.

##### Scripts

The first script (*programmingBasics.m*) provides basics of data file import and data manipulation, including accessing and appending values into data arrays as well as calculating basic features of behavioral temporal data. Two additional scripts provide basics for plotting and modifying simple event and timeseries data (*timeseriesBasics.m* and *eventDataBasics.m*). These scripts allow users to view behavioral data with simple plots and provide syntax for common modifications of color and line specification and finishing touches for axes and titles. They also introduce users to techniques for summarizing and combining data streams, as well as “for loops” for cycling through arrays. All three scripts are designed to be run one line at a time, with scripted material designed to showcase various types of manipulations and their outputs, as detailed in the *readme.md* file and the inline documentation in scripts. Additionally, the first two scripts contain practice problems with solutions to challenge the user to begin independently modifying scripts.

The fourth script guides the users through the process of importing and transforming event-coded data from common annotation software. Outputs from annotation software typically contain both numerical and text data which are difficult to import using common file read functions. The *annotationImport.m* script provides code to transform outputs of annotation software into a clean event-data format that can be easily manipulated and accessed in Matlab and will be used in Module 2. The fifth and sixth scripts, *convertEvents2Timeseries.m* and *convertTimeSeries2Events.m*, offer codes to convert imported event data sequences into timeseries format and timeseries data into event format temporal data. Finally, the script *convertEvents2Binaryspikes.m* can be used to convert event data exported by the *annotationImport.m* function into binary spike train data. These scripts thus allow researchers to more easily transform their own input data for use in subsequent modules, as well as other potential applications.

## Module 2: Getting to Know Your Data: Visualization of High-Density Multi-Modal Interactions

This module introduces more complex techniques for visualizing behavioral data streams. With advances in video and sensing technology, it is increasingly possible for researchers use high-density multidimensional data to gain insight into the real-time processes of behavior (de Barbaro, 2019). For example, researchers interested in understanding of early emotion regulation could annotate—or potentially automatically detect markers of—frame-by-frame changes in mother and child affect, gaze, and patterns of touching to examine real-time strategies mothers utilize to regulate children’s distress and their impacts on subsequent soothing (see, e.g., Ye et al., 2012; Kim and Clements, 2015; de Barbaro et al., n.d.). These annotations could be further synchronized with heart rate or electrical brain signals (de Barbaro et al., 2017; Wass et al., 2019) to examine concurrent physiological regulation, or to assess whether individual differences in physiology might moderate the impacts of mothers’ regulation efforts.

Data visualization can highlight the structure of such complex behavioral processes within and between participants, providing researchers key insights throughout the analytical process (Gnisci et al., 2008). In early stages of analysis, visualizations of raw or minimally processed data can provide insights into underlying patterns and regularities. Critically, the novelty of high-density multimodal datasets means social scientists have limited vocabulary and insight into these data at such high levels of granularity. In this case, summarizing data using prespecified measures can be misleading, and has the potential to overlook the most relevant or interesting features of the data. By providing access to raw data for inspection, visualizations can highlight temporal or multi-modal structure that may not be specified by existing theory (Yu et al., 2012).

In later stages of analysis, data visualization can ensure the validity and quality of operationalizations of phenomena of interest, as well as help to interpret observed results. For example, one useful approach for working with high-density datasets is to identify repeated “events” occurring within the data stream (de Barbaro et al., 2013; Granic and Hollenstein, 2016). Such events can help to parse the unfolding interaction into manageable and relevant instances of behavior. If events are derived from raw data, marking their position within the timeline of raw data can help to ensure valid and meaningful definition of event boundaries. Additionally, iterative visualization of raw data with increasingly processed data can indicate the density and temporal ordering of events relative to other data streams, helping to guide the selection of relevant analytic techniques.

### Methods

Creating intuitive and meaningful visualizations involves a number of methodological considerations. Collected temporal data may include multiple channels, each representing a different dimension or modality of behavior, each with potentially distinct properties. For example, researchers may want to combine multiple channels of temporal data representing behaviors that are binary, such as the presence or absence of joint gaze, with data that have many different mutually exclusive categories, such as qualitatively distinct emotions or dyadic states, or continuous data, such as physiological signals, or affect levels ranked from more negative to more positive. Color, positioning, and line style can be used to represent these different types of activity to most intuitively highlight the structure of multimodal behavior. For example, qualitatively distinct activities may be better represented with different colors whereas continuous affect may be best represented via a timeseries. Alternatively, the structure of continuous affect data may be best revealed by parsing continuous affect data into “positive,” “negative,” and “neutral” categories. Ultimately, these decisions are made through a mixture of theory, intuition, and simple trial and error.

Overall, visualization of high-density multimodal data often requires flexibility not present in off-the-shelf visualization tools included with data collection or annotation software. Scripting visualizations affords infinite control over these decisions, ultimately allowing customizable exploration of data structure. The current module thus showcases three sets of scripts used to visualize complex multimodal data collected by developmental scientists. The datasets span multi-participant event data, physiological data synchronized with event data, and multiple synchronized channels of physiological activity from a wearable sensor. Finally, the scripts in this module allow batch processing of multiple study participants, facilitating within- and between-participant comparisons. We recommend beginning with visualizations of 6–10 study participants to access the structure and variability of your data, increasing this number if saturation is not apparent.

#### Sample Data and Scripts

This module builds upon the basic data manipulation and plotting techniques provided in Module 1 to provide readers with experience visualizing more complex multimodal and multi-participant behavioral data. The visualization module includes sample data from three different datasets, three main scripts including a demo script *demo_visualizations*, and a *readme.md* file that provides instructions for running each script. The demo script can be used to create a number of appealing plots of high-density multimodal behavioral data.

##### Data

To provide users experience plotting a variety of different types of data streams, three sample datasets are provided: (1) frame-by-frame mother-and-infant affect data in a sample of mothers with a history of depression (Lusby et al., 2014; Goodman et al., 2017), (2) infant heart rate data collected over the course of a laboratory session that includes attention and learning tasks presented on a monitor (de Barbaro et al., 2016a, 2017) and (3) pilot data of the author of the module wearing a wrist-mounted physiological sensor that collects heart rate, electrodermal activity and motion on a day that she gave a department seminar. To use these scripts on their own data, users will need to have data formatted as timeseries and/or events using the scripts *annotationImport.m* and *convertEvents2Timeseries.m* in Module 1.

##### Scripts

The three scripts in this module provide the user with practical visualization techniques for multimodal datasets. The script *multiParticipantEventPlotting.m* plots three dimensions of mother and infant affect into a single plot, distinguishing positive, neutral, and negative affect via intuitive color and vertical positioning. The *plotTimeseriesWithEvents.m* script combines synchronized timeseries (infant heart rate) and event-data (tasks) into a single plot to provide insight into potential relations between infants’ activities and physiological changes. Finally, the script *plotSensorData.m* script plots three different types of wearable physiological data in three plots on a single figure, to indicate the temporal relations between these measures. This script processes Unix timestamps, a specialized time format commonly used by sensor platforms.

### Results

**FIGURE 1:**
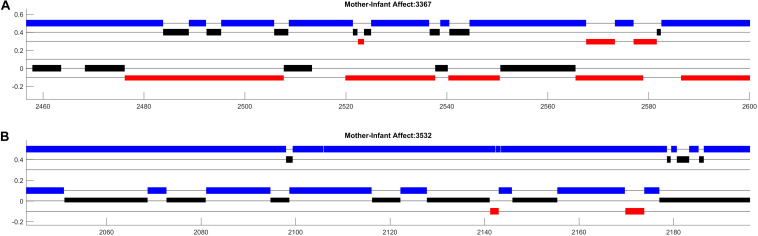
Affect data from two mother–infant dyads participating in a face-to-face free play session at 3 months of age. The *x*-axis shows time (in seconds) and the *y*-axis distinguishes different dimensions of affect. Maternal affect is represented in three rows at the top **(A)**, infant affect is represented in three lower rows **(B)**. For both mothers and infants, the highest of the three rows (in blue) represent positive affect, the middle row (in black) represents neutral affect, and the bottom row (in red) represents negative affect.

**FIGURE 2:**
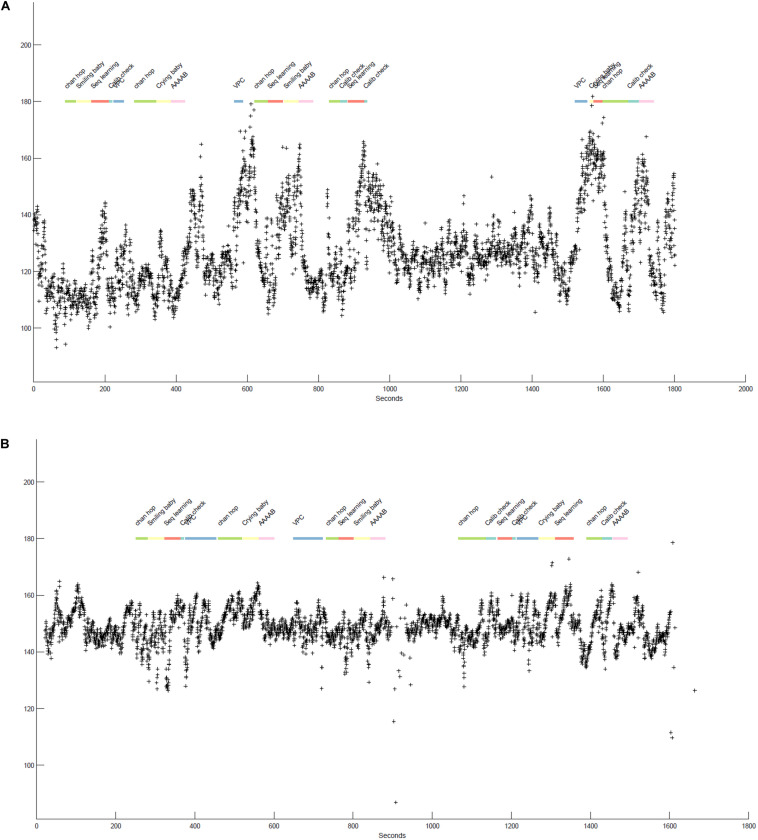
**(A,B)** illustrate the beat-by-beat heart rate data (indicated by + symbols) of two infants participating in various tasks over the course of a laboratory session. The *x*-axis shows time (in seconds) and the *y*-axis specifies the heart rate values in equivalent beats-per-minute. The colorful line at the top represent the specific tasks infants participated in, differentiated by color and labels. The boundaries of each segment indicate the start and stop times of each task.

To provide examples of the types of insights that visualizations can provide, we will walk through the figures generated by the scripts *multiParticipantEventPlotting.m* (Figure 1) and *plotTimeseriesWithEvents.m* (Figure 2).

Figure 1 displays approximately 150 s worth of dynamic affect data from two mother–infant dyads participating in a face-to-face free play session at 3 months of age. While the color and positioning parameters of this image could be arbitrarily changed, setting these parameters in an intuitive way can greatly facilitate the comprehension of your plots (Tufte, 2001). In this example, higher position on the *y*-axis corresponds to more positive affect, the color red indicates distress, and colors are consistent across mother and infant affect states. Additionally, the simple black lines (“kebab lines”) help to orient the observer to where data could be present. Finally, setting the width and height of the bars such that that each dimension of mother and infant affect is “touching” highlights potential contingency between mother and infant affect.

Organized in this way, the plots highlight salient aspects of interaction that can be examined further in systematic analyses. First, in both dyads we observe that both mothers and infants’ cycle between different affect states across the session. Additionally, there appear to be many instances of contingent affect shifts between mothers and their infants. Note, for example, that each shift in affect displayed by the mother in dyad 3532 (Figure 1B) appears to be contingent on a shift in infants’ affect. Perhaps most strikingly, we observe strong differences in the expression of negative affect between the two infants, such that one infant displays less than 10 s of negative affect while the other displays nearly 10 times that. Visualizations of additional participants (not shown) indicated high levels of infant negative affect expression in 10–20% of mother–infant interactions. This led us to consider that there was large variation in the challenge faced by mothers in responding contingently to their infants which may moderate the relations between contingent affect responding and infants’ affect development longitudinally. It also led us to wonder whether patterns of maternal activity were contributing to these variations in infant affect, given that caregiver sensitivity is often associated with infant negative affect expression. We are exploring these questions in ongoing research (de Barbaro et al., 2020).

Figure 2 displays approximately 25–30 min of two 12-month-old infants’ heart rate data as they participate in different tasks during a laboratory session. Together, the plots highlight individual differences in infant heart-rate reactivity as well as the presence of task-associated changes in heart rate. Differences in heart rate reactivity appear stable within each infant across tasks, i.e., Figure 2A consistently shows strong responses to tasks with increases in heart rate ranging from 20to 80, while Figure 2B shows much more moderate task-associated increases in heart rate, with heart rate typically increasing by 20–30 beats from task onset.

Neither infant shows noticeable session-level effects, that is, heart rate increases are generally followed by a return to some sort of “baseline.” However, where there is a gap between tasks (e.g., between 1,000 and 1,600 s for Figure 2A), both infants heart rate is meaningfully lower and more stable, again suggesting that the tasks themselves are arousing or potentially stressful for the infants. Finally, the stretch of low heart rate in both infants at the start of the task, encompassing the “chan hop” and “smiling baby” tasks, suggested that this segment of the session might function as a valid baseline. We followed up on the generated insights in multiple manuscripts. For example, we examined individual differences in heart rate reactivity to the visual paired comparison (VPC; habituation) task and their association with performance on this task (de Barbaro et al., 2016a). We also examined how the changes in heart rate across the session were associated with changes in attention as assessed by eye-tracker gaze duration (not plotted here; see de Barbaro et al., 2017).

### Discussion

As Bakeman and Quera (2011) note, sequential analyses are not “off the shelf.” No single analytic tool can characterize dense, multi-channel behavior dynamics of social interaction. Instead, investigators should anticipate an iterative process to converge on the analytic tools that will capture the temporal structure of their data (de Barbaro et al., 2013). Visualizations of high-density behavioral data can prove critical in this process. In particular, data visualized in an intuitive way can highlight salient aspects of the temporal structure of activity and thereby guide the selection of relevant analytic techniques.

## Module 3: Tapping Into the Temporal Structure of Developmental Data Using Burstiness Analysis

**FIGURE 3:**
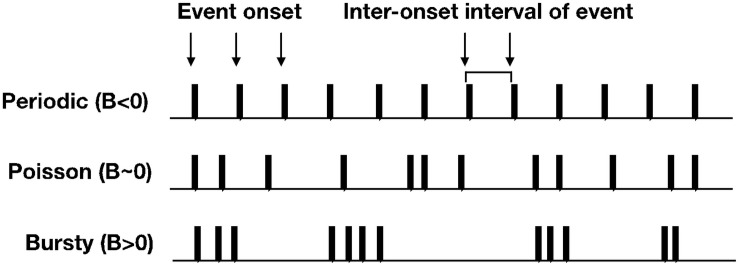
Toy examples of spike trains that approximate periodic, Poisson, and bursty temporal structure.

In this section, we will introduce methodological advancements for how developmental scientists can apply simple distributional analyses to time series data to estimate the temporal structure of event-level datasets. The burstiness analysis can be useful for psychologists interested in studying the temporal patterns of behavior. For example, the ability to quantify and/or categorize temporal patterns of behavior using a simple metric, can lead to generating and testing hypotheses about how a particular behavior unfolds over time. This analysis, first introduced in statistical physics (Goh and Barabási, 2008), characterizes spike trains of human behavior in the dimension of burstiness of the spike train of interest. Burstiness is a distributional measure that provides an estimate of a system’s activity patterns spanning from periodic (−1 < *B* < 0), to random (*B* ∼ 0), to theoretically maximal burstiness (0 < *B* < 1) (see Figure 3).

### Methods

As noted above, the analysis requires the user to have a binary spike train of 0 s and 1s in which a ‘1’ represents the onset of an event of interest and a ‘0’ represents instances when an onset did not occur. Inter-onset intervals are computed from the binary spike train and then the inter-onset interval distribution is submitted to an estimation of burstiness. A simple equation provides an estimation of burstiness with the assumption of an infinite time series,

$$B=\frac{\sigma_{\tau }-\mu_{\tau }}{\sigma_{\tau }+\mu_{\tau }}$$

where σ_τ_ is the standard deviation of the inter-onset interval distribution and μ_τ_ is the mean of the inter-onset interval distribution. A recent addition to the burstiness analysis includes an updated equation that takes into account the amount of inter-onset intervals in a distribution and therefore is more relevant for empirical work using finite time series (Kim and Jo, 2016). Estimates from both equations converge when the inter-onset interval distributions include τ_length_ > 100 intervals.

#### Sample Data and Scripts

The burstiness module contains two samples of data, two scripts including a demo file that quantifies and visualizes burstiness across different data streams, and a *readme.md* file that provides step-by-step instructions for running the scripts.

##### Data

Example data come from a randomly selected subject in a developmental study where ego-centric views were collected at a sample rate of 1/5 Hz in infant’s natural environments (see Jayaraman et al., 2015, 2017; Fausey et al., 2016; Jayaraman and Smith, 2019). Human coders coded each frame for the presence of hands or faces in the field of view. Sample data include two spike train series, one for when a hand came into view and one for when a face came into view. To use this script on their own data, users will need to have data formatted as binary spike trains, which they can do using the *convertEvents2Binaryspikes.m* script in Module 1. Additionally, the user should be aware of the sampling rate that was used to collect and process the data as this will constrain interpretations of the magnitudes of inter-onset intervals.

##### Scripts

**FIGURE 4:**
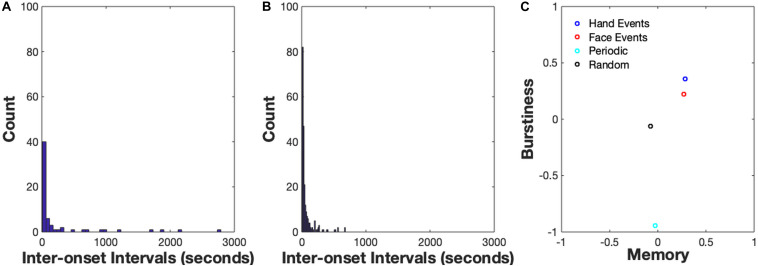
**(A)** Inter-onset Interval Distribution for hand events, **(B)** Inter-onset Interval Distribution for face events. An IOI distribution for a periodic signal (*B* < 0), the user would observe high counts of IOIs in one particular timescale, e.g., a high amount of IOIs with length = 10 s. An IOI distribution for a bursty signal (*B* > 0) is typically right-skewed, suggesting a high frequency of short IOIs and less-frequent (but non-zero) amount of longer IOIs. **(C)** Burstiness-Memory plot. On the *x*-axis is a measure of the memory of the event series which is commonly estimated by the lag-1 autocorrelation coefficient.

The demo script *demo_bursty.m* calculates the burstiness of the two example spike trains. It also generates a periodic spike train and a random spike train generated from a Poisson process and calculates the burstiness of those data streams to provide a comparison for the burstiness values of the sample data. Finally, it generates a plot (Figure 4) to provide a visual comparison of the burstiness of each spike train as well as a distribution of the inter-onset intervals of the sample data streams. Additional details are provided in the script in-line documentation.

### Results

The results from the *demo_bursty* script are shown in Figure 4. The first two images display the distribution of inter-onset-intervals of the two sample data streams. The IOI distribution for a periodic signal (*B* < 0) would indicate high counts of IOIs at one particular timescale, e.g., a high amount of IOIs with length = 10 s. By contrast, an IOI distribution for a bursty signal (*B* > 0) is typically right-skewed, suggesting a high frequency of short IOIs and less-frequent (but non-zero) amount of longer IOIs. Estimates of Burstiness, *B*, are indicated along the *y*-axis of the right-most image in the plot. The *x*-axis is a measure of the memory of the event series which is commonly estimated by the lag-1 autocorrelation coefficient. Due to the introductory nature of this module, we won’t discuss the concept of memory in detail here, please see Goh and Barabási (2008) for a comprehensive explanation. Note that the Burstiness values of the empirical spike trains are both positive, meaning they are both in the ‘bursty’ regime as opposed to the ‘random’ or ‘periodic’ regime: *B* = 0.22 for the Face spike train and *B* = 0.36 for the Hand spike train. The two burstiness estimates suggest that bouts of hand activity are more clustered in time and have longer periods of time when hand activity was not occurring relative to bouts of face activity. Observing that the hand events are more bursty than the face events also suggests that bouts of manual activity are likely occurring at faster timescales compared to face events coming in and out of the infant’s view. Moreover, observing that both the hand events and face events are not organized in a periodic or random temporal structure suggests a more complex collection of constraints acting on the two information sources, such as social interactions, toy play, etc. Finally, the burstiness estimate of the randomly distributed events is approximately 0 and the burstiness of events generated from the periodic distribution is slightly less than −1, as would be expected of these distributions.

### Discussion

The burstiness analysis affords the researcher with the ability to provide a simple index of the temporal structure of an event of interest. One critical limitation of the application of the burstiness analysis to a wide range of behavioral event series is the relative magnitude of the *B* estimate across datasets. This limitation suggests that the user should use caution when directly comparing *B* estimates across datasets. One strategy that has been used in recent research that applied the burstiness analysis to multimodal human interaction (Abney et al., 2018), was to generate bootstrapped confidence intervals to determine categorical boundaries of periodic, random, and bursty temporal structure. For example, a Poisson process is generated by an interevent interval distribution of an exponential distribution. Therefore, a researcher can generate confidence intervals by first simulating a sample of interevent interval distributions with similar properties to the empirical dataset (e.g., average size of interevent interval distribution) but from an exponential distribution and then estimate the lower and upper bound of the confidence intervals of what the burstiness analysis would suggest would be as Poisson (*B* ∼ 0). Generating bootstrapped confidence intervals of the lower and upper bounds of what the burstiness analysis would classify as ‘Poisson’ can allow a researcher to then classify empirical spike trains with known burstiness values. Although this strategy allows the researcher to classify behavioral events into intuitive categories of temporal structure, the main limitation of this strategy is that classification does not afford the researcher to develop, test, and reject hypotheses about the magnitude of *B* estimates and cognitive mechanisms. Despite the important limitations the user should consider, the burstiness analysis provides a simple metric that can inform researchers about the temporal structure of behaviors of interest.

## Module 4: Cross-Recurrence Quantification Analysis of Dyadic Interaction

The techniques introduced in this section are variations of Recurrence Quantification Analysis (RQA) (Marwan et al., 2007), which is a powerful non-linear time-series technique originating from the natural sciences, and which has gained popularity in the social sciences in the past two decades. RQA can be applied to data of a continuous and nominal measurement level, and to a single data stream as well as to a pair of data streams. The latter version of the technique is called Cross-Recurrence Quantification Analysis (CRQA) (Shockley et al., 2002). In this module we will focus on two recent advancements for nominal data streams, known as Chromatic CRQA and Anisotropic CRQA (Cox et al., 2016), which are particularly suited for the analysis of differentiation and asymmetry in dyadic interaction. This choice is given by two observations: the first is that many datasets in psychology originate from annotations of audio or video recordings, from manual or automated registration of gaze across regions of interest (ROI), or from a comparable procedure resulting in temporally ordered sequences of distinct behavioral categories (i.e., nominal event or time-series data). The second is that many research questions pertain to social interaction, in which a number of people, typically a dyad, are engaged in some form of interpersonal behavior, for instance, two children collaborating on a task (Guevara et al., 2017) or a mother–infant feeding interaction (van Dijk et al., 2018).

CRQA enables researchers to study attunement and coordination in such dyads. Dyadic interaction typically consists of recurrent patterns of several types of matching and non-matching (i.e., collective) behaviors of the interaction partners. These patterns can be of various duration and are potentially coupled across different episodes of the interaction. That is, interaction partners might influence each other’s behavior (almost) immediately, but this influence might also be delayed for some shorter or longer period of time. Also, dyadic attunement is sometimes brief and consists of only a single behavioral category, but it might also be a lengthier behavioral sequence consisting of several different categories. CRQA detects such behavioral patterns and quantifies their dynamic characteristics and temporal associations. In addition, one may wish to track the different types collective behaviors of the dyad separately, and weigh the relative contribution of each of the interaction partners to the recurrent patterns. Chromatic and Anisotropic CRQA facilitate this. It can reveal temporal structure in data streams across different timescales, which remains inaccessible to most other time-series methods. There are several good texts explaining CRQA for continuous and nominal time series emphasizing conceptual issues and applications (Webber and Zbilut, 2005; Marwan et al., 2007; Wijnants et al., 2012b). In the following sub-sections, we will introduce the technical underpinnings of Chromatic and Anisotropic CRQA, detail the key scripts in our code modules, and explain the derived measures and the features of Chromatic and Anisotropic CRQA with an example.

### Methods

**FIGURE 5:**
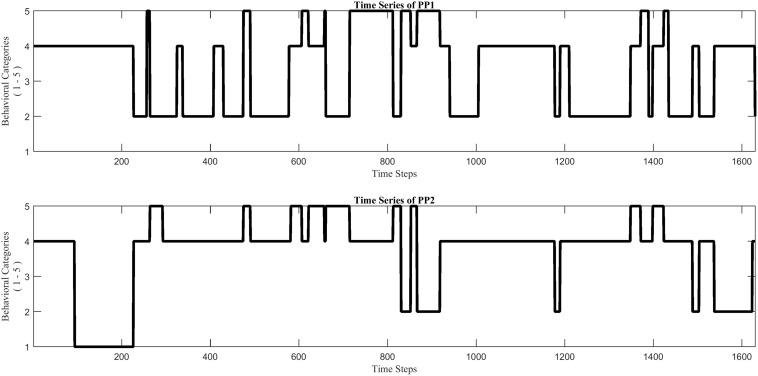
Two nominal time series (*example_data.mat*), each of which reflects the stream of collaborative behaviors of a child during a dyadic interaction, coded by using the same five specific behavioral categories (1–5) for each.

The central feature of CRQA is the Cross-Recurrence Plot (CRP; Figure 5), which visualizes the temporal organization of the interaction based on ‘recurrences.’ What counts as a recurrence is pre-defined by the researcher, and in its most general form it can be any matching pair of individual behaviors of the two interaction partners (e.g., shared gaze, both lifting, as well as complementary “matches” such as speak-silence, offering-accepting). For nominal data a CRP is easily constructed by tracking such behavioral matches across the entire lengths (*N*) of the two data streams. By holding one of the data streams along the horizontal axis and the other along the vertical axis, the occurrences of behavioral matches are plotted in the two-dimensional CRP with *N* rows ad *N* columns. Each dot in the CRP represent the instance where a behavioral state of the horizontally presented interaction partner is matched in a specific way by that of the vertically presented one. When several different types of behavioral matches (i.e., qualitatively different combinations of behavioral categories) need to be tracked simultaneously, this can be represented in the CRP with a color coding. This is represented in Figure 5, where you can see two colors, representing two types of behavioral match (the white areas are the remaining non-matching states). This version of the method is called *Chromatic CRQA* (see Cox et al., 2016).

The distribution of the dots across the CRP captures the interactional dynamics, and several measures can be derived to quantify this. The simplest measure is the recurrence rate (*RR*), which is the proportion of behavioral matches. *RR* provides a crude measure of coordination between the two interaction partners across multiple timescales. Note that individual dots are no longer visible in Figure 5, because they tend to constitute smaller and larger rectangular patterns. These are quite common in a CRP of nominal data coming from dyadic interaction, and reflect both a persistence in the interaction as well as the coarse-grained nature of the measurement (Cox et al., 2016; Xu and Yu, 2016). A rectangular pattern indicates an episode of behavioral-category use of one interaction partner which was accompanied by some episode of matching behavioral-category use of the other interaction partner. The more asymmetric the pattern, the briefer the matching behavior is performed by one interaction partner compared to the other. The overall asymmetry (or rather: anisotropy) in the CRP is therefore related to asymmetries in the dynamics, and provides information about differences in the relative contribution and dominance between the interaction partners.

Given the rectangular structure and the possible anisotropy of the CRP, it makes sense to quantify the horizontal and vertical extent of the behavioral patterns separately and analyze the differences between the two orientations. This version of the method is called *Anisotropic CRQA* (Cox et al., 2016). The CRQA measures in the CRQA module quantify the proportion of patterns, their mean and maximum length, and their entropy, for both orientations. Specifically the measures are: (1) *LAM* (Laminarity), which is the proportion of matches constituting patterns in the vertical and horizontal orientation, (2) *TT* (Trapping Time), which is the average length of vertical and horizontal patterns, (3) *Max_L*, which is the length of the longest vertical and horizontal pattern, and (4) *Ent_L*, which is the Shannon entropy of vertical and horizontal length distribution. Note that each of these measures can be calculated for every type of behavioral match (i.e., color in the CRP) separately. Given the introductory nature of this module, the four measures we covered in this module are a subset of CRQA measures. This subset of measures are chosen since they are among the most widely used CRQA measures by studies from various disciplines (Fusaroli et al., 2014). They are especially relevant for the analysis of nominal data streams of dyadic interaction (as argued in Cox et al., 2016), which are often collected in developmental studies. For additional measures and software to calculate them, please see (Webber and Zbilut, 2005; Marwan et al., 2007; Coco and Dale, 2014; Hasselman, 2018).

#### Sample Data and Scripts

The CRQA module contains a readme.md file, an example dataset, and six MATLAB scripts, including a demo function *demo_CRQA.m.* These materials will enable users with little to no programming experience to plot the data and perform simple Chromatic and Anisotropic CRQA.

##### Data

The example dataset *example_data.mat* consists of two nominal time series, PP1 and PP2, of equal length (1,630 time steps), each containing integer values from 1 to 5. The time series come from a dyadic interaction study, in which the collaborative behaviors of two children were coded from video, at 1 Hz, by using the same five specific behavioral categories for each (for more details see Guevara et al., 2017). To use this script on their own data, users will need to have data formatted as timeseries, which they can do using the *convertEvents2timeseries.m* script in Module 1.

##### Scripts

The function *demo_CRQA.m* loads the example data and runs the entire set of functions of the module. The function *tt.m* in the folder *lib* is part of the crp toolbox available at http://www.recurrence-plot.tk, and computes the distributions of lengths of the vertical and horizontal line structures in the CRP. Based on these two distributions, the orientation-specific CRQA measures (*LAM*, *TT*, *Max_L*, and *Ent_L*) are calculated. The CRQA module can be performed directly on the example dataset, but with a few modifications of the scripts also on the user’s own nominal dataset.

**FIGURE 6:**
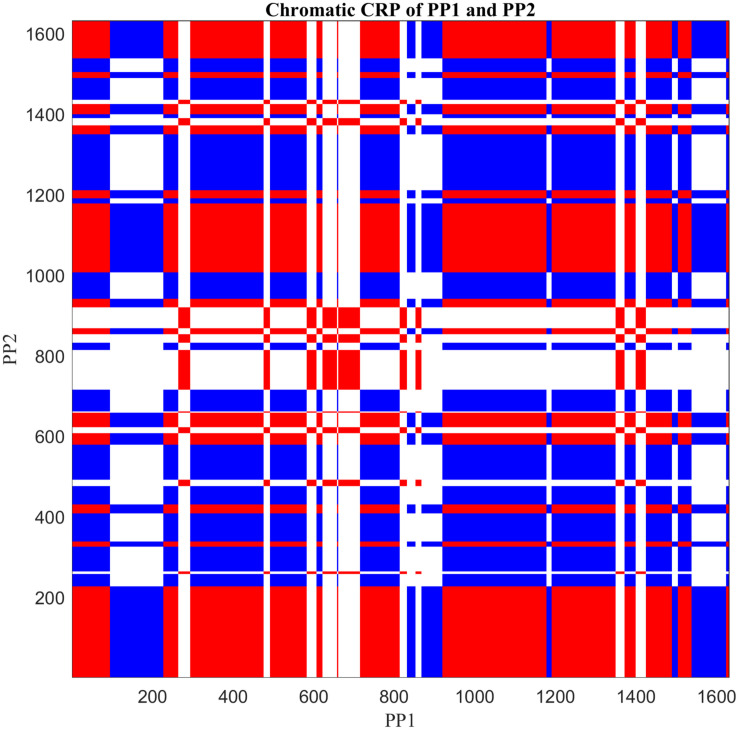
Cross-recurrence plot of the nominal time series shown in Figure 4, by applying Chromatic CRQA. The three colors represent three different types of collaborative states of the dyad. (For details see text).

The function *PlotTS.m* visualizes the two time series. This opens a figure window showing two plots, each of which displays the behavioral stream of one of the interaction partners (Figure 5). The function *CatCRMatrix.m* creates a cross-recurrence matrix *rec* of the two time series. Two types of behavioral matches are distinguished in this analysis: ‘distributed dyadic interaction’ (DDI) and ‘unequal dyadic interaction’ (UDI). In DDI both children were actively engaged with the task and contributed to the solution, whereas in UDI only one child was contributing to the solution while the other child was not (see Guevara et al., 2017 for more details). All other combinations of individual behaviors are considered to be non-matching, and were labeled ‘no dyadic interaction’ (NDI). Different values in the matrix *rec* correspond to different types of behavioral matches, +1 for DDI and −1 for UDI, whereas NDI receives the value 0. The function *PlotCRP.m* plots the Chromatic CRP, based in the matrix *rec* (see Figure 6). The function *CRQA_out.m* performs Chromatic and Anisotropic CRQA using the matrix *rec*. Chromatic CRQA calculates the recurrence rate (*RR*) for both types of behavioral matches. The function provides *RR* both as a proportion of the total number of points in the CRP (i.e., canonical recurrence rate), as well as a proportion of the total number of behavioral matches (i.e., relative recurrence rate). These values are written to the matrix *Chromatic_CRQA* in the Workspace (Table 1). Anisotropic CRQA quantifies both the vertical and horizontal patterns. As said, the quantitative analysis will ignore the different types of behavioral matches for now, treating them as equal. The orientation-specific CRQA measures are written to the matrix *Anisotropic_CRQA* in the Workspace (Table 2). The upper row in *Anisotropic_CRQA* gives the values for the vertical line structures, the lower row those for the horizontal line structures.

**TABLE 1:** Canonical Recurrence Rate (*RR*) and relative Recurrence Rate (*rRR*) for both types of behavioral match after Chromatic CRQA.

***Matching type***	***RR***	***rRR***
DDI (red)	0.31	0.46
UDI (blue)	0.36	0.54

**TABLE 2:** CRQA measures of the horizontal and vertical patterns after Anisotropic CRQA.

***Patterns***	***LAM***	***TT***	***Max_L***	***Ent_L***
Vertical	1.00	104	453	3.05
Horizontal	1.00	98	482	3.05

### Results

Figure 6 displays a colored checkerboard pattern typical for Chromatic CRQA with nominal data. There are three colors in this CRP representing the three different types of states of the dyad, based on the numerical values in *rec*: red for DDI (value +1), blue for UDI (value −1), and white for NDI (value 0). All together the CRP nicely displays the rich coordinative structure of the collaborative interaction, across all possible timescales, in terms of the pre-defined behavioral matches. The values in *Chromatic_CRQA* show differences in the recurrence rate for DDI and UDI. Specifically, the recurrence rate for UDI is slightly higher than for DDI. This means that UDI is the dominant attractor state, implying that the unequal dyadic interactions were more prominent than the distributed dyadic interactions, in this particular interaction. The values in *Anisotropic_CRQA* (Table 2) quantify the patterns of the vertical and horizontal line structures displayed in the Figure. Notably, we see small differences between some of the CRQA measures for the horizontal and vertical patterns, reflecting a small anisotropy in the CRP. *LAM* is equal to 1 for both orientations, which means that all recurrences are part of a horizontal pattern as well as a vertical pattern (of at least length 2). In general terms this means that the collaborative behavior is quite patterned. *TT* is slightly higher in the vertical direction than in the horizontal direction. This is reversed for *Max_L*. This is also somewhat visible in Figure 5. Finally, there is no difference between *Ent_L* for both orientations. Overall, these results indicate an asymmetry in the dynamics of this interaction, suggesting slight differences in the behavioral dominance between the two collaborating children. These results are investigated further in Guevara et al. (2017).

### Discussion

Although the asymmetry in the example data is relatively small, additional examples of the explanatory and predictive value of Chromatic and Anisotropic CRQA are available in the literature (e.g., De Jonge-Hoekstra et al., 2016; Guevara et al., 2017; López-Pérez et al., 2017; Nonaka and Goldfield, 2018; Menninga et al., 2019; Gampe et al., 2020). For reasons of simplicity the module materials do not allow users to assess the quantitative recurrence metrics for different types of behavioral matches (i.e., the different colors displayed in the CRP). Chromatic CRQA on nominal data can result in a large number of measures. That is, for each behavioral match included in the analysis there is an additional set of measures. This even becomes almost twice as large when quantifying the line structures for both orientations in the CRP, as is done in Anisotropic CRQA. However, the relative differences of the anisotropic CRQA measures can quantify relevant asymmetries in the dyadic dynamics and the differences in coupling strength between two interaction partners (see, e.g., Cox et al., 2016) and thus provides a valuable addition to toolbox of the high-density behavioral analyses.

## Module 5: Discovering Directional Influence Among Multimodal Behavioral Variables With Granger Causality

In this section, we will introduce Granger Causality (GC), a method to quantify the directional influences among a set of interdependent behavioral variables. Wiener–Granger Causality is a statistical notion of causality based on computing the improved prediction from one time series to another (Granger, 1969; Bressler and Seth, 2011). Consider an infant–parent toy-play interaction as a concrete example. In this interaction, multiple behavioral cues from both the infant and the parent are influencing each other at the same time. GC could be used to examine the directional influence from one specific behavior to another in this multimodal interaction. For example, with GC we can compute whether a parent talking about a toy increases the likelihood of the child looking at that same toy in real time, above and beyond all the other behavioral variables observed in this social interaction.

Originally developed in the context of econometric theory, GC gained popularity within the field of neuroscience as a non-invasive technique for inferring relations in between different sources of neural activity (Roebroeck et al., 2005; Vakorin et al., 2007; Chang et al., 2008; David et al., 2008; Nedungadi et al., 2009). Recently, GC has also been used in behavioral research. For example, it has been used to examine the early development of vocal turn-taking between marmoset monkey infants and parents (Takahashi et al., 2016), to quantify leader and follower dynamics in joint music performance (Chang et al., 2017) and to examine the development of coordinated behaviors in infant–parent interaction (Xu et al., 2017). In the following subsections, we explain the conceptual foundation of this technique and then demonstrate how to calculate and interpret the results with an empirical example from an infant–parent interaction study.

### Methods

Wiener (1956) provided the conceptual basis of Granger causality, namely, the idea that the variable *X* could be said to cause *Y* if the ability to predict *Y* can be improved by incorporating the information contained in *X*. Granger (1969) formalized this notion of causality in the domain of time series signals based on multivariate autoregressive (MVAR) models. The basic idea of MVAR is quite simple: the past can predict the future. For example, the behavior of a complex system *H* at time *T* + *1* can be modeled by its past observed behaviors or values from *T − p* to *T*. Granger causality can be described within this example as follows. Suppose that *X* and *Y* are two interdependent processes in this system *H* and we want to predict the future of *Y*. First, we predict *Y*’s value at time *T+1*, i.e., *Y*_*T+1*_, using all the available information in the system *H* from time *T − p* to *T*, including its own past values (i.e., its history) and the history of *X*. Next, we calculate a second prediction for *Y*_*T+1*_, this time using all the available information in the system *H* from time *T − p* to *T*, including its own history, but this time, excluding the past values of *X*. If *Y*_*T+1*_ is better predicted in the model that includes the past values of *X*, this means that past values of *X* contain unique information that helps to predict *Y* above and beyond the information contained in the history of all the other variables, *Y* itself included. In this case, *X* is said to Granger-cause *Y*.

Currently, GC can be applied to time series with continuous values or binary spike trains (see Figure 3) introduced in Modules 1 and 2. Binary spike trains are used to indicate whether or not a given activity, such as a neuron firing or a child’s babbling is observed during a sampling period. The MVGC Matlab Toolbox developed by Barnett and Seth is widely used for computing GC among time series with continuous values. Barnett and Seth (2014) offers a comprehensive tutorial covering both the mathematical foundation of the GC’s computational process and the usage of the MVGC toolbox. For this reason, this module focuses on the computation of GC within discrete binary spike train data based on the framework and toolbox developed by Kim et al. (2011).

For discrete binary spike trains or point processes, the likelihood of an event occurring is modeled by a Generalized Linear Model (GLM): a linear combination of this temporal variable’s dependency to the history of each individual element in the ensemble. The GLM framework allows researchers to calculate the statistical significance of the GC relationship using the likelihood ratio test statistic. These goodness-of-fit statistics can be calculated by comparing the deviance between the estimated model with trigger variable *X* excluded and the estimated full model in the GLM framework. Additionally, a multiple hypothesis testing error measure, the false discovery rate (FDR) (Benjamini and Hochberg, 1995; Storey, 2002) can be used to assess the expected proportion of FDR when the number of hypothesis tests and thus the number of rejected null hypotheses is large.

#### Sample Data and Scripts

The fifth code module contains all the Matlab functions involved in the calculation of GC and its significance, two sample data files, a demo file, *demo_granger_causality.m*, which demonstrates all steps of GC calculation with the provided data, and a *readme.md* file, which provides detailed instructions on how to use the scripts.

##### Data

Our example dataset includes multimodal behavioral streams collected from an infant–parent toy-play experiment. Infant and parent dyads participated in a toy-play experiment when the infant was 9- and 12-months old. During each visit, the dyads were instructed to play with three single colored toys as they would if they were playing at home. Both participants wore head-mounted eye-trackers to record their eye movements (Franchak et al., 2011) and their first-person view of the play episode. An additional birds-eye camera captured their manual activities from above. The parent’s speech was also recorded. After the experiment, eye-tracking data, video recordings and speech recordings were synchronized and calibrated. Trained coders provided frame-by-frame annotations indicating all instances of parents and infants gaze and manual contact with each of the three objects (Slone et al., 2018). Three ROI were used for all the behavioral streams: the three toys. In addition, we transcribed the parent’s speech data, and identified all instances during which an object’s name was referenced and marked those naming events with ROI values as well. In summary, we collected five behavioral time series: infant gaze, infant manual activity, parent gaze, parent manual activity, and parent speech.

**FIGURE 7:**
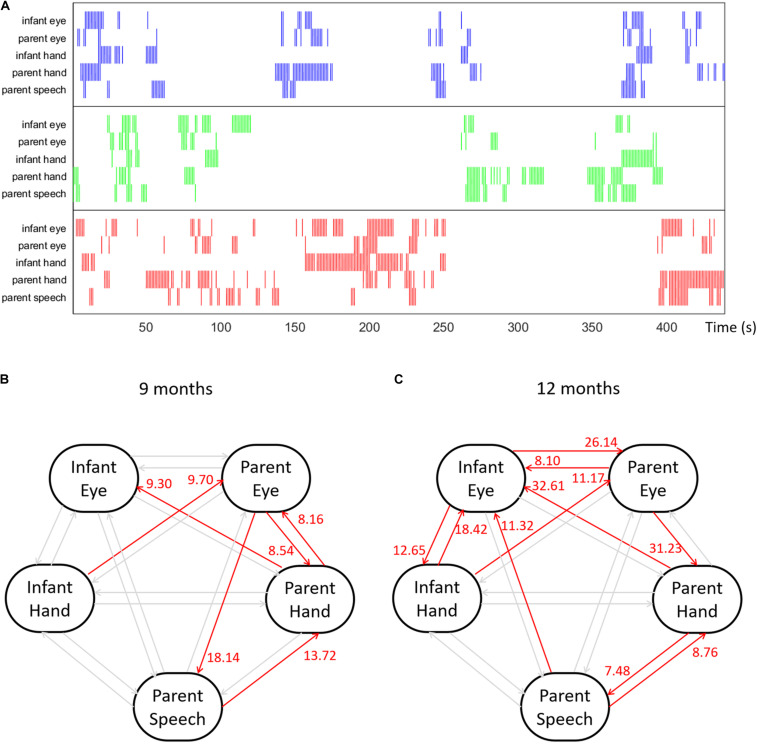
**(A)** An example multimodal temporal dataset collected from an infant–parent interaction study with five behavioral variables: infant gaze behavior, infant manual action, parent gaze behavior, parent manual action and parent speech events with object names. The colors indicate the target object of each behavior. **(B,C)** Granger causality results computed among five behavioral time series for an example dyad at 9 and 12 months.

To convert our behavioral data streams into multivariate spike train data, all data streams were divided into three groups according to each object, i.e., the ROIs. Next, at every 333 ms interval we re-sampled the behavioral streams to see if each behavior was present during the interval. For example, if the infant looked to the red object during a given sample unit, this section was marked 1; if not, 0. After resampling, the behavioral streams were transformed into spike trains. Figure 7A shows a portion of visualized raw behavioral streams from our sample dataset. Data file *gcause_sample_data1.mat* contains the behavioral streams collected from the example dyad when the infant was 9 months; file *gcause_sample_data2.mat* contains the behavioral streams collected from the same dyad when the infant was 12 months. To use this script on their own data, users will need to have data formatted as binary spike trains, which they can do using the *convertEvents2Binaryspikes.m* script in Module 1.

##### Scripts

The demo script *demo_granger_causality.m*, loads one of the sample datasets, visualizes the behavioral time series in a plot (see Figure 7A for an example), performs GC computation and displays the results in an easily interpretable format. The key function for computing GC and conducting significance test is *calculate_granger_causality()* which is located in the *lib/*folder. This function takes two input parameters: *data_matrix* and *glm_time_range*. The first parameter, *data_matrix*, contains the time series data that will be used to perform GC computation. The second parameter, *glm_time_range*, is the length of the history window that will be used for prediction model fitting in GC computation.

In the calculation, the function *calculate_granger_causality()* will first generate a set of likelihood estimation models for each time series contained in *data_matrix* by iterating through history window durations from 1 to *glm_time_range*. The best estimation will be chosen from this set of candidate models using Akaike’s information criterion (AIC) (Akaike, 1974; Burnham and Anderson, 1998). Next, the function will calculate GC from between every pair of variables. For example, in order to calculate the extent to which the infant looking at the red object uniquely improves the occurrence of the parent also looking at the red object, the function constructs two models: (1) the full model: the likelihood of parent’s looking behavior is modeled based on the recent history of all five variables in our system; (2) the partial model: the function excludes infant’s looking variable from the model and calculates an estimate of the parent’s looking behavior based on the other four variables alone.

The results are returned in the output *results_gcause_mat*. The magnitude indicates the strength of the GC influence: higher value means stronger influence. The second return value *results_gcause_fdr* contains the significance test result for every directional GC influence. The significance test can result in three outputs: 1, −1, or 0. An output of 1 means that there is a significantly positive GC directional influence between the indicated pair of variables; −1 means that there is a significant negative GC influence; and a 0 value means the influence is not significant. Lastly, the function *prettyprint_gcause_result()* will print out the quantified directional links among each pair of variables in an easily readable format in the console.

### Results

Figures 7B,C shows the Granger Causality results among five behavioral variables for one example dyad at 9 and 12 months. With five behavioral time series, we computed twenty different types of directional links between each pair of variables. Figure 7B shows a visualized graph illustrating the computed Granger causality links among five behavioral variables of the example dyad when the infant was 9-months old; Figure 7C shows the computed result of the same dyad at 12 months. In the Figure, red links indicate the significantly positive links with number near the arrow of each link representing the G-cause value. In this example, at both ages, this dyad shows a significantly positive link from the parent’s manual action to infant’s looking behavior. This means that the occurrence of parent’s holding a certain object significantly increases the likelihood of the infant looking at the same object, i.e., parent’s manual actions Granger caused infant’s looking behavior in infant–parent interactions when the infant was 9 and 12 months. Note that the influence from parent to child increases across this developmental period. We can also see that influences between infant modalities also increase between 9 and 12 months. Thus, using GC techniques, we observed developmental changes in the directional influences between parent and child multimodal (gaze hand and parent speech) activity.

### Discussion

The fact that Granger Causality accommodates stochastic processes and makes only general assumptions about the collected data means it is particularly well-suited for behavioral scientists who collect multiple dimensions of behavior each mutually influencing one another. However, we note a number of limitations of this technique. First, it currently lacks the flexibility for behavioral data collected from trials with different lengths. Second, the trigger variable must occur before the effect variable in the behavioral data coding in order to be featured as a predictor in the GC computational process. Methods of data recording or coding that lack precise temporal accuracy may thus obscure “granger-causes.’ Finally, while behaviors and their mutual influences can be non-linear, the current modeling process is based on a linear assumption: all causal influences remain constant in direction throughout time (Granger, 1988; Sugihara et al., 2012; Maziarz, 2015). Addressing these limitations would enhance the application of GC to complex behavioral data.

## Overall Discussion

From Lashley (1951) to Elman (1990) and Kolodny and Edelman (2015), understanding the temporal structure of human behavior has been recognized as one of the most fundamental problems in psychology. Recently, advances in technology have allowed us to collect large datasets of high-density behavioral streams in naturalistic scenarios (de Barbaro, 2019). This has enabled researchers to capture the temporal dynamics of behavior at a fine temporal scale. Additionally, it has created novel analytical challenges for modern psychologists. To tackle these challenges, we provide introductions and scripts for a range of complementary analysis techniques. For the novice programmer, we provide a guide to the basic functions required for time series analysis (Module 1). Data visualization techniques introduced in Module 2 allow users to create flexible and customizable plots of raw and processed behavioral data streams to provide insight into structure and variability of behavior within and between participants. In the third module, we introduce Burstiness calculations to describe the overall distributional structure and quantify the occurrence regularities of temporal events. In the fourth module, we explain Chromatic and Anisotropic Cross-Recurrence Quantification Analysis, which quantify non-linear dynamics across multiple timescales. These methods can track different types of recurrent behavioral patterns within dyadic interaction and can quantify asymmetries in the dominance between interaction partners. Finally, we introduce Granger causality techniques (Module 5) to quantify the directional relations among multiple interdependent behavioral variables within a system. Each module includes sample data collected by developmental psychologists, and scripts provided in Module 1 allow users to import and format their own data for use in subsequent modules. Experienced programmers can modify scripts as desired.

It would be impossible to provide a complete introduction to all techniques relevant to multimodal high-density data. Many other techniques capture temporal dependencies in sequential data, including but not limited to Markov-chain based graphical modeling (Pentland and Liu, 1999), fractal (Scafetta and Grigolini, 2002; Chen et al., 2010; Wijnants et al., 2012a) and multifractal analyses (Ihlen, 2012; Kelty-Stephen et al., 2013), dynamic field modeling (Thelen et al., 2001; Cox and Smitsman, 2019), and dynamic causal modeling (Stephen and Mirman, 2010). Such formal modeling approaches hold assumptions about the structure and mechanisms of activity. The approaches introduced here are mainly descriptive, making at most minimal assumptions about the structure of the input data and therefore naturally accommodating stochastic processes.

Technology and novel computational methods are changing the landscape of the field of psychology. Although considerable effort has been devoted to open science and data sharing (MacWhinney, 2014; Gilmore et al., 2016; Foster and Deardorff, 2017; Frank et al., 2017), researchers often neglect “method sharing.” As pointed out by Caiafa and Pestilli (2017), in this new data-intensive era, effectively every experiment is the convergence of three key dimensions: *data*, *analytics*, and *computing*. Platforms such as OpenNeuro (Gorgolewski et al., 2017) and brainlife (Hayashi et al., 2017; Avesani et al., 2019) have successfully achieved this vision of sharing data, analysis methods and computing resources in neuroscience. As psychologists begin to grapple with novel big-data techniques for studying behavior, similar platforms could unite researchers with different expertise to enhance scientific communication and discovery while reducing cost of conducting novel and interdisciplinary research. By sharing real data with detailed code for a variety of techniques for analyzing high-density multimodal activity, we take a first step toward the new behavioral science of tomorrow.

## Data Availability Statement

Sample datasets used in this paper are provided at: https://github.com/findstructureintime/Time-Series-Analysis.

## Ethics Statement

The studies involving human participants were reviewed and approved by Institutional Review Board (IRB) at Human Subjects Office Indiana University. Written informed consent to participate in this study was provided by the participants’ legal guardian/next of kin.

## Author Contributions

TX, KB, DA, and RC contributed to conception of this article. TX and KB contributed equally to the main body of the manuscript as co-first authors. KB wrote the text and scripts of Modules 1 and 2. DA wrote the text and scripts of Module 3. RC wrote the text and scripts of Module 4. TX wrote the text and scripts of Module 5. All authors contributed to the article and approved the submitted version.

## Conflict of Interest

The authors declare that the research was conducted in the absence of any commercial or financial relationships that could be construed as a potential conflict of interest.
